# Death from Aconite—Inquest

**Published:** 1845-10

**Authors:** 


### *Death from Aconite : Inquest.*

The death of a respected member of the profession, Dr. Male, was announced in a late number of the Provincial Journal. In consequence of various rumors which had been circulated as to the cause of this event, an inquest was held, of which the following account, taken from the Birmingham Advertiser of July 31, presents some points of interest connected with the exhibition of powerful medicines :—

The first witness, John Barker, deposed that he had lived in the service of the deceased nearly eight years, and spoke to the doctor's health up to a recent period. He was 66 years of age. On Wednesday, the 23d, he was out, and on Thursday morning, about seven o'clock, witness took him up a cup of coffee, which he drank. He (witness) then went to the stable, and soon after deceased came down complaining of a pain between the bowels and the chest. Deceased had complained for some time past of pains in the back. He had some warm water taken up to him, and was sick two or three times. Deceased asked him if he looked unwell, and witness replied he looked very unwell. In a short time, Miss Male, his daughter, came to him about nine o'clock. On Friday the deceased sent for him; he appeared alarmingly ill, more so than he had ever seen him before. He said he wished to bid him, (witness,) farewell, and to evince his kindness and respect towards him. He seemed to consider himself in a dying state. He did not blame any person for his illness. Witness did not know of his taking any medicine.

Mr. Russell, surgeon, of Newhall Street, was next sworn, and said, I have known the deceased for a number of years. He has complained to me occasionally, for six weeks or two months past, of pains in the back and loins. On Thursday morning, about half-past nine, his son, the Rev. Dr. Male, came for me, and on going to his father's house, I found the deceased in bed. His extremities were cold, the general surface of the skin cold and clammy, the pulse quick and feeble, (at 130,) with cramps and pains in his legs, and spasmodic pains in his stomach. He said his head was confused. He told me that, not experiencing relief, (alluding to the pains,) from medicines in ordinary use, he had been taking tincture of aconite. He then asked me if I had ever given the medicine, and I said no. I then asked him what doses he had taken, and he replied on the preceding Sunday five drops, two or three times a day. I cannot be positive whether he said twice or thrice, but I believe he said two or three times a day, and had increased it to six, eight and ten drops. One dose of ten drops only had been taken on the previous night. He had been also suffering from diarrhœa for a few days, and he had taken a dose of ten drops of solution of opium early that morning for it. I inquired where he had got his notion from relative to the aconite. He said he had been reading a book now circulating through our societies treating upon the advantage of aconite in similar pains. He expressed his conviction that he should die, that the medicine was too powerful for him; but he also expressed his most earnest desire that he might recover, as his life was of the utmost importance to his children at this time. This he repeated during his illness, to myself and Dr. James Johnstone. I cheered him as much as I could, reminding him of his former depression when ill, and that I thought he had nervous power sufficient to wear out the effect of the medicine he had taken. I gave him mild aperients to overcome the poison, with camphor and ammonia. His son-in-law, Mr. Amphlett, saw him along with me in the evening, and we left him somewhat, in our opinion, relieved.—On Friday we again met, and towards evening with Dr. James Johnstone, as we found him more sunk. Dr. Johnstone agreed with us in our treatment, &c. Late that evening I found him in a dying state, gradually sinking. He was in a torpid state, from which, however, he could easily be roused, and then his intellects were clear. He had no paralysis. His death took place about ten o'clock on Saturday morning. He was perfectly composed, and took an affectionate leave of myself and others, reminding me that for thirty-five years we had lived together in an uninterrupted friendship. Twenty hours after death I made a *post-mortem* examination, in the presence of Mr. Clayton, Dr. James Johnstone, Dr. Bell Fletcher, and my son. His body, with the slightest possible exception, was in a healthy state. The blood was unusually fluid. Witness described the appearances, which presented nothing remarkable, and concluded by attributing death to the accumulated doses of the aconite depressing the nervous system.

In answer to some questions put by jurors,

Mr. Russell said that such doses would not be likely to leave traces in a *post-mortem* examination beyond a fluidity in the blood. Had deceased been a younger man, in all probability he would have recovered from the shock of the medicine. Aconite is little used, and he was not prepared to say that ten drops would produce fatal effects.

The Coroner then briefly alluded to the circumstances which had induced the family to request an inquiry into the melancholy event, an event which he, in common with the medical profession generally, most sincerely deplored. The deceased was respected and honoured in life, and his loss so calamitously brought about, would be extensively and deeply felt by more than one class of society.

The jury then gave in a verdict of "Accidental death from an overdose of aconite taken medicinally by the deceased."—*Provincial Med. & Surg. Journal.*

